# Combined effects of aging and inflammation on renin-angiotensin system mediate mitochondrial dysfunction and phenotypic changes in cardiomyopathies

**DOI:** 10.18632/oncotarget.3979

**Published:** 2015-05-18

**Authors:** Tyesha N. Burks, Ruth Marx, Laura Powell, Jasma Rucker, Djahida Bedja, Elisa Heacock, Barbara J. Smith, D. Brian Foster, David Kass, Brian O'Rourke, Jeremy D. Walston, Peter M. Abadir

**Affiliations:** ^1^ Division of Geriatric Medicine and Gerontology, Johns Hopkins University School of Medicine Baltimore, MD 21205, USA; ^2^ Division of Cardiology, Johns Hopkins University School of Medicine Baltimore, MD 21205, USA; ^3^ Cell Biology Imaging Facility, Johns Hopkins University School of Medicine Baltimore, MD 21205, USA

**Keywords:** aging, mitochondria, AT1R, inflammation, heart

## Abstract

Although the effects of aging and inflammation on the health of the cardiac muscle are well documented, the combined effects of aging and chronic inflammation on cardiac muscle are largely unknown. The renin-angiotensin system (RAS) has been linked independently to both aging and inflammation, but is understudied in the context of their collective effect. Thus, we investigated localized cardiac angiotensin II type I and type II receptors (AT_1_R, AT_2_R), downstream effectors, and phenotypic outcomes using mouse models of the combination of aging and inflammation and compared it to a model of aging and a model of inflammation. We show molecular distinction in the combined effect of aging and inflammation as compared to each independently. The combination maintained an increased AT_1_R:AT_2_R and expression of Nox2 and exhibited the lowest activity of antioxidants. Despite signaling pathway differences, the combined effect shared phenotypic similarities with aging including oxidative damage, fibrosis, and hypertrophy. These phenotypic similarities have dubbed inflammatory conditions as premature aging, but they are, in fact, molecularly distinct. Moreover, treatment with an AT_1_R blocker, losartan, selectively reversed the signaling changes and ameliorated adverse phenotypic effects in the combination of aging and inflammation as well as each independently.

## INTRODUCTION

Age-related changes in the cardiovascular system lead to a cardiomyopathy characterized by left ventricular hypertrophy and fibrosis [1]. When untreated, this cardiomyopathy can lead to heart failure, one of many cardiovascular diseases (CVDs). CVDs account for approximately 80% of deaths in individuals over 65 years old making it the primary cause of age-related deaths in the United States of America [2]; thus aging is a major risk factor of CVDs. Another risk factor of CVDs is inflammatory conditions such as rheumatoid arthritis. Individuals with inflammatory diseases are disproportionately impacted by CVDs and may develop a phenotype that is consistent with age-related cardiomyopathy [3]. These phenotypic similarities have led to inflammatory conditions being classified as premature or accelerated aging. Moreover, individuals with inflammatory conditions are at a higher risk of being classified as frail [4]. Frailty is a syndrome characterized by multiple factors including increased inflammatory markers and decreased body mass and functional parameters that confers high risks for morbidity and mortality [5–7]. With individuals over 65 years of age being the fastest growing age group [8] and the increased risk of frailty on CVDs [9], it is important to investigate the interplay between aging and chronic inflammation on the pathogenesis and progression of cardiomyopathies.

The process of aging is associated with chronic, low-grade inflammation characterized by increased levels of cytokines including interleukin-6 (IL-6) [6, 10]. Evidence suggests that age-related, low-grade systemic inflammation develops in otherwise healthy individuals possibly as early as the age of 55 years old [11]. Evidence also suggests that changes in mitochondria are influenced by chronic inflammation, and as a result, the increased free radical production from dysfunctional mitochondria further activate chronic inflammation creating a vicious cycle. Over time and due to the lack of proper defense mechanisms, this positive feedback loop leads to exacerbation of oxidative damage, reduction in ATP production, loss of cardiomyocytes, and formation of fibrotic tissue [12, 13]. It is also important to note that the accumulation of damaged mitochondria typically appears in humans by mid to late 70s; yet, the early mitochondrial changes in healthy subjects predate the accumulation of damaged mitochondria by decades in otherwise healthy people [14].

Analogous features between aging and inflammatory diseases include systemic inflammation, elevated oxidative stress and mitochondrial dysfunction; all of which have also been implicated in the pathogenesis of age-related cardiomyopathies [3, 15]. However, what activates and governs the onset and the rate of progression of these molecular changes is still unclear. One of the hormonal factors that has been independently linked to the progression of aging and inflammatory conditions is the renin-angiotensin system (RAS) [16, 17]. RAS acts mainly through two receptor subtypes, angiotensin II type 1 and type 2 (AT_1_R and AT_2_R) that in general lead to opposing actions upon stimulation [16]. The unregulated binding of angiotensin II (AngII) to AT_1_R has been linked to many deleterious effects including oxidative stress, inflammatory pathway activation, and mitochondrial dysfunction [18, 19]. Additionally, increased AT_1_R signaling has been shown to induce pathological cardiac remodeling that has been reversed with AT_1_R blockers (ARBs) [20]. The primary cardio-protective benefits of ARBs, such as losartan, are believed to arise from systemic effects on blood pressure and cardiovascular remodeling. However, a local, independently-regulated cardiac RAS exists and may play a role in the progression of cardiomyopathies [16] and the benefits of ARBs.

Here we sought to investigate the progression and development of age-related cardiomyopathies in the pre-existing context of inflammatory conditions. We focus on cardiac RAS in chronic inflammation preceding aging (combination of aging and inflammation) and how it compares to aging preceding inflammation and inflammation (without the process of aging). Moreover, we are interested in the reversibility of pathological findings using an ARB. To model inflammation and the combination of aging and inflammation, we utilized a mouse deficient for interleukin-10 (IL-10), an anti-inflammatory cytokine, that has been used in aging studies to model chronic inflammation and frailty [21-23]. The lack of IL-10 causes increased expression of nuclear factor-kappa B-induced inflammatory mediators [22, 24]. Furthermore, these mice are characterized by elevated serum levels of several cytokines including IL-6 [21, 22] which has been associated with inflammatory conditions [25] and adverse outcomes in humans [6, 10].

Our data demonstrates that aging, inflammation and the combined effect of both are all characterized by aberrant AT_1_R signaling, but they exhibit unique downstream effectors. Aging and the combined effect culminated in mitochondrial dysfunction and cardiac decompensation. Furthermore, losartan treatment altered the aberrant signaling and ameliorated some of the downstream effects.

## RESULTS

To ascertain the influence of aging, inflammation, and the combined effect on local cardiac RAS, downstream effectors and cardiac health, we compared young and aged C57BL/6J wild-type (WT) mice to age- and gender-matched IL-10 knockout (IL-10^−/−^) mice. Aged WT mice represent aging, young IL-10^−/−^ mice represent inflammation and aged IL-10^−/−^ mice represent the combined effect.

### Changes in cardiac renin-angiotensin signaling

**Figure 1:**
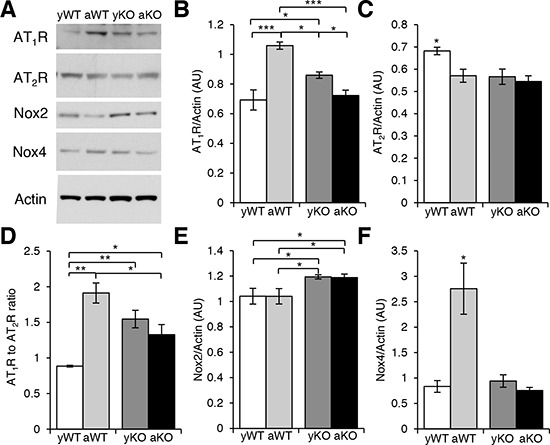
Expression of localized RAS receptors and downstream effectors in cardiac muscle **A.** Western blot analyses of cardiac protein extracts from young WT (yWT), aged WT (aWT), young IL-10^−/−^ (yKO) and aged IL-10^−/−^ (aKO) mice using antibodies against angiotensin II type 1 (AT_1_R) and type 2 (AT_2_R) receptors and NAPDH oxidases (Nox) 2 and 4. Actin was used as a loading control. **B. C. E. F.** Relative expression was calculated for the Western blots displayed in (A.) in arbitrary units (AU). **D.** Ratio of the relative expression of AT_1_R (B.) to AT_2_R (C.). Data are means ± SEM. *p < 0.05; **p < 0.01; ***p > 0.001.

RAS is a key hormonal system whose dysregulation has been linked to aging, inflammation, mitochondrial dysfunction, and CVDs. We show that the expression of AT_1_R was highest in the cardiac muscle of the aged WT and increased in the young IL-10^−/−^ mice as compared to the young WT and the aged IL-10^−/−^ mice (Figure 1A–1B). Interestingly, the combined effect of aging and inflammation (aged IL-10^−/−^) decreased the expression of AT_1_R as compared to the aged WT mice (Figure 1B) which was unexpected given that AT_1_R levels correlate with negative outcomes in chronic inflammation [26]. In contrast, the levels of AT_2_R were similarly decreased in the aged WT, young IL-10^−/−^ and aged IL-10^−/−^ as compared to young WT mice (Figure 1A, 1C).

Given that AngII binds with equal affinity to AT_1_R and AT_2_R [16] suggesting an important role of the ratio between AngII receptors [27], we calculated the ratio of cardiac AT_1_R to AT_2_R (AT_1_R:AT_2_R ). There was an increased AT_1_R:AT_2_R in the aged WT, young IL-10^−/−^, and aged IL-10^−/−^ mice as compared to the young WT (Figure 1D). There was also an increase in the AT_1_R:AT_2_R in aged WT as compared to aged IL-10^−/−^ mice consistent with the AT_1_R levels (Figure 1B). While aging, inflammation and the combination had differential effects on the expression levels of AT_1_R, our data suggests a consistent increase in AT_1_R:AT_2_R under all of these conditions and supports the notion that it may contribute to the pathogenesis of cardiomyopathies.

One of the many deleterious effects of AT_1_R signaling is the production of reactive oxygen species (ROS), which can be initiated through the activation of NADPH oxidase (Nox). In the cardiac muscle, there are two predominant isoforms of Nox, Nox2 and Nox4, which are the major sources of superoxide anion (O_2_ ) and hydrogen peroxide (H_2_O_2_) [28]. Nox2 levels were increased in the young and aged IL-10^−/−^ mice (Figure 1A, 1E). In contrast, Nox4 was only increased in the aged WT mice (Figure 1A, 1F). This data implies differential expression of Nox proteins due to age or inflammation: Nox2 is up-regulated with chronic systemic inflammation and Nox4 is up-regulated with ‘general’ aging suggesting distinct downstream signaling effectors of increased AT_1_R:AT_2_R ratio and/or effects of alternate activators of Nox [29].

### Increased free radical damage in the cardiac muscle

**Figure 2:**
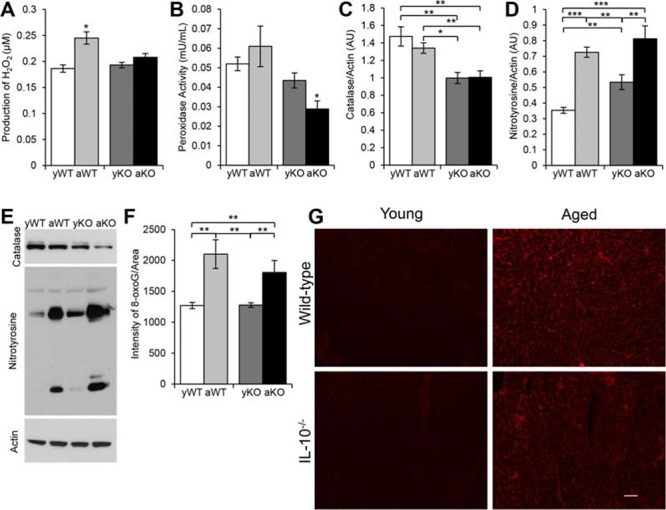
Measurements of reactive oxygen species, antioxidants and oxidative damage in cardiac muscle **A.** Amount of H_2_O_2_ produced by mitochondria isolated from cardiac muscle of young WT (yWT), aged WT (aWT), young IL-10^−/−^ (yKO) and aged IL-10^−/−^ (aKO). **B.** Peroxidase activity measured in mitochondria isolated from cardiac muscles. **C–D.** Relative expression for the Western blot analyses of cardiac protein extracts **E.** using an antibody to catalase and nitrotyrosine in arbitrary units (AU). Actin was used as the loading control. **F.** Quantification of fluorescent intensity of 8-oxoguanine (8-oxoG) immunostaining **G.** of cardiac muscle cryosections. Scale bar: 100 μm. Data are means ± SEM. *p < 0.05; **p < 0.01; ***p < 0.001.

We next determined the redox balance in aging, inflammation, and the combination. The highest levels of mitochondrial ROS (H_2_O_2_) were present in the aged WT mice (Figure 2A).

Regardless of age, catalase levels were reduced in the IL-10^−/−^ mice (Figure 2C, 2E). Moreover, the lowest activity of peroxidase was seen in the combination of aging and inflammation (aged IL-10^−/−^) (Figure 2B). Due to the distinction in ROS generation and antioxidant levels, we examined the cardiac muscle for oxidative damage. There was an increase in DNA damage in the aged WT and IL-10^−/−^ mice compared to their respective younger counterparts (Figure 2F, 2G). Furthermore, we investigated reactive nitrogen species-induced damage. Nitrotyrosine levels increased with age, inflammation, and the combination. The aged WT and aged IL-10^−/−^ mice had more nitrosylated proteins than the younger mice, and the young IL-10^−/−^ had more than the young WT mice (Figure 2D–2E). These results indicate that the burden of oxidative stress results from increased ROS production in ‘general’ aging and a decrease in antioxidant levels during inflammation.

### Impaired cardiac mitochondrial function and structure

**Figure 3:**
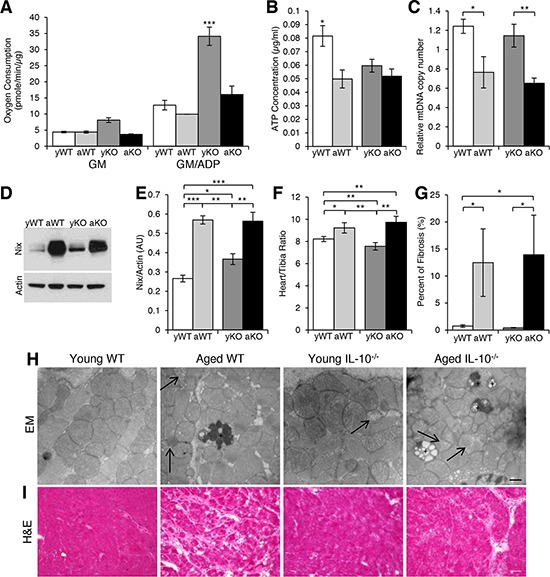
Altered mitochondrial parameters and cardiac remodeling **A.** Oxygen consumption from mitochondria isolated from cardiac muscle of young WT (yWT), aged WT (aWT), young IL-10^−/−^ (yKO) and aged IL-10^−/−^ (aKO) after the addition of glutamate/malate (GM) in State 4 and GM and ADP in State 3 respiration. **B.** ATP concentration in cardiac muscle. **C.** Relative mitochondria DNA (mtDNA) copy number as a measure of the ratio between cytochrome B and GapDH. **D.** Western blot analyses using an antibody against Nix and **E.** the relative expression in arbitrary units (AU). Actin was used as a loading control. **F.** Cardiac hypertrophy measured as a ratio of heart weight to tibia length. **G.** Quantification of fibrosis evident in (I.). **H.** Representative EM pictures of mitochondria in cardiac muscle. Arrow shows depleted mitochondria. “+” is labeling lipofuscin granulas. Scale bar: 500 nm. **I.** Histological analyses of cardiac muscle cryosections using H&E. Scale bar: 100 μm. Data are means ± SEM. *p < 0.05; **p < 0.01; ***p < 0.001.

The high-energy cardiac muscle is composed of a large amount of mitochondria and susceptible to oxidative damage. Therefore, we investigated the structure and function of cardiac mitochondria under conditions of aging, inflammation, and the combination. Interestingly, the mitochondria from the young IL-10^−/−^ mice had the highest state 3 oxygen consumption levels (GM/ADP) of all the groups (Figure 3A). Furthermore, when we compared the percent difference with age between the WT and IL-10^−/−^ mice, the decrease in oxygen consumption in the IL-10^−/−^ mice was almost four-fold the decrease in WT mice (−112.5% vs. −28.3%). We then measured cellular ATP concentration, which was the highest in the young WT mice (Figure 3B). The dissociation between oxygen consumption capacity and net ATP levels may potentially be explained by differences in ATP utilization and/or the amount of dysfunctional mitochondria. Indeed, there was evidence of damaged mitochondria present in the aged WT, young IL-10^−/−^ and aged IL-10^−/−^ mice (Figure 3H).

The turnover of old and damaged mitochondria is crucial to the health and function of the cardiac muscle. Nix is a protein marker expressed on damaged mitochondria and required for autophagic induction [30]. There was an age-related increase in Nix levels in both genotypes, but also higher Nix expression in the young IL-10^−/−^as compared to the young WT mice (Figure 3D–3E) indicating the presence of damaged mitochondria as evident in Figure 3H. The age-related increase in Nix-marked mitochondria was associated with an age-related decrease in the relative mitochondria DNA (mtDNA) copy number (Figure 3C), which has been proposed to be a measure of mitochondrial function [31]. This decline in the number and/or function of the mitochondria with aging, inflammation and the combination may play a role in the pathogenesis of cardiomyopathies.

### Measurements of pathological cardiac remodeling

The excess AT_1_R signaling, ROS production, and dysfunctional mitochondria observed during aging, inflammation, and the combination subjects the cardiac muscle to chronic stress, which may lead to cardiac remodeling. The process of pathological cardiac remodeling includes many functional and structural changes including hypertrophy and interstitial fibrosis [28]. There was age-related cardiac hypertrophy and fibrosis in both the aged WT and IL-10^−/−^ mice (Figure 3F, 3G, 3I). The absence of hypertrophy and fibrosis in the inflammation model (young IL-10^−/−^) would support a causal role for the process of aging in the development of this cardiac phenotype.

### Treatment with ARBs to counteract the effects of excess AT_1_R signaling

Due to the hypothesized involvement of increased AT_1_R contributing to the observed cardiac pathology, we sought to determine the impact of blocking AT_1_R on the cross-talk between RAS, mitochondrial health and cardiac remodeling in aging, inflammation, and the combination. We omitted young WT from treatment because there was no increase in the AT_1_R:AT_2_R nor evidence of cardiomyopathy development.

**Figure 4:**
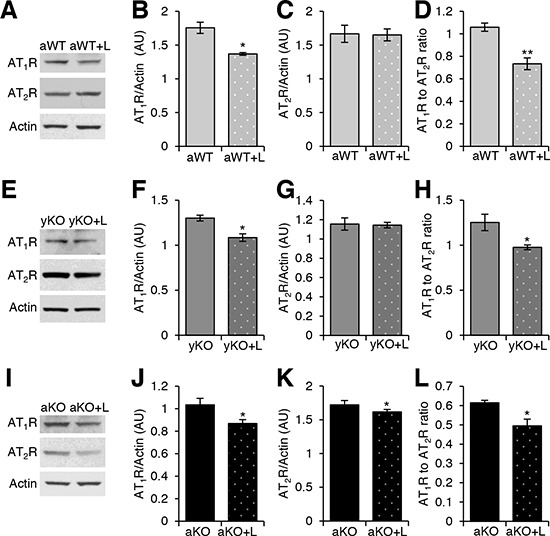
Losartan treatment modulated the cardiac renin-angiotensin system receptors Western blot analyses of cardiac protein extracts from aged WT (aWT) **A.**, young IL-10^−/−^ (yKO) **E.**, and aged IL-10^−/−^ (aKO) **I.** mice with (+L) or without Losartan treatment using antibodies against angiotensin II type 1 (AT_1_R) and type 2 (AT_2_R) receptors. Actin was used as a loading control. Relative expression was calculated for the Western blots displayed in arbitrary units (AU) for aWT **B–C.**, yKO **F–G.** and aKO **J–K**. Ratio of the relative expression of AT_1_R to AT_2_R for aWT **D.**, yKO **H.** and aKO **L.**. Data are means ± SEM. *p < 0.05; **p < 0.01.

**Figure 5:**
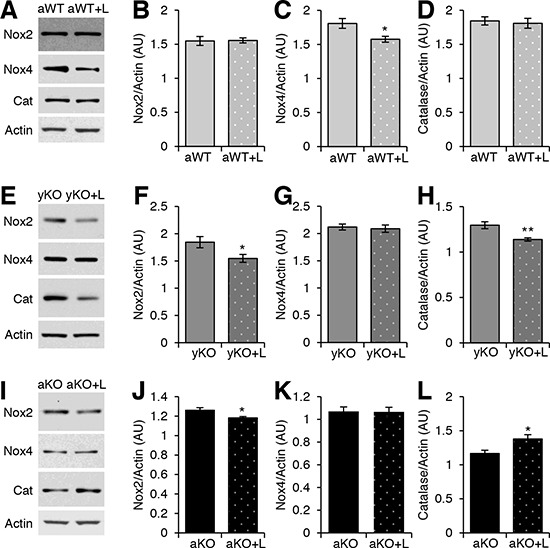
Effects of losartan treatment on downstream effectors of AT1R and antioxidants Western blot analyses of cardiac protein extracts from aged WT (aWT) **A.**, young IL-10^−/−^ (yKO) **E.**, and aged IL-10^−/−^ (aKO) **I.** mice with (+L) or without losartan treatment using antibodies against NADPH oxidases (Nox) 2 and 4 and Catalase (Cat). Actin was used as a loading control. Relative expression was calculated for the Western blots displayed in arbitrary units (AU) for aWT **B–D.**, yKO **F–H.** and aKO **J–L**. Data are means ± SEM. *p < 0.05, **p < 0.01.

Losartan treatment (Los) reduced the expression levels of AT_1_R in the aged WT (Figure 4A–4B), young IL-10^−/−^(Figure 4E–4F), and aged IL-10^−/−^ (Figure 4I–4J) mice as compared to their respective placebo-treated, age- and genotype-matched counterparts. While Los did not change the AT_2_R expression in aged WT (Figure 4A, 4C) and young IL-10^−/−^ mice (Figure 4E, 4G), it decreased AT_2_R expression in the aged IL-10^−/−^ mice (Figure 4I, 4K). Nonetheless, the AT_1_R:AT_2_R was decreased in all losartan-treated groups as compared to their placebo-treated counterparts (Figure 4D, 4H, 4L). We next measured the effects of Los on the downstream effectors of AT_1_R. Los decreased Nox2 levels in the young (Figure 5E–5F) and old (Figure 5I–5J) IL-10^−/−^ mice, but had no effect on levels in aged WT (Figure 5A–5B). Levels of Nox4 were decreased in the aged WT (Figure 5A, 5C), but unchanged in the young (Figure 5E, 5G) and aged IL-10^−/−^ mice (Figure 5I, 5K) with treatment. Los had a differential impact on the downstream effectors by decreasing the specific Nox proteins that were differentially increased in aging versus inflammation.

### Rectification of molecular, phenotypic and functional changes with ARB treatment

**Figure 6:**
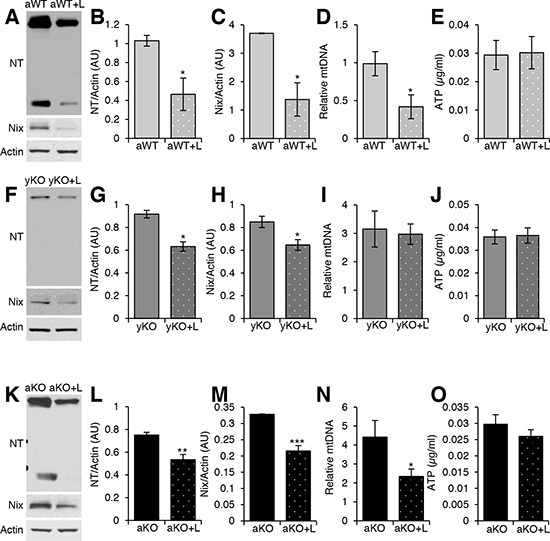
Measurements of oxidative damage and mitochondrial health after treatment with AT1R blocker Western blot analyses of cardiac protein extracts from aged WT (aWT) **A.**, young IL-10^−/−^ (yKO) **F.**, and aged IL-10^−/−^ (aKO) **K.** mice with (+L) or without losartan treatment using antibodies against Nitrotyrosine (NT) and Nix. Actin was used as a loading control. Relative expression was calculated for the Western blots displayed in arbitrary units (AU) for aWT **B–C.**, yKO **G–H.** and aKO **L–M.** Relative mitochondria DNA (mtDNA) copy number as a measure of the ratio between cytochrome B and GapDH for aWT **D.**, yKO **I.** and aKO **N.** ATP concentration of cardiac muscle of aWT **E.**, yKO **J.** and aKO **O.** Data are means ± SEM. *p < 0.05; **p < 0.01; ***p < 0.001.

Since treatment with losartan reduced the levels of AT_1_R, we investigated its effects on oxidative stress and mitochondrial health. Catalase expression was increased in the aged IL-10^−/−^ (Figure 5I, 5L), unchanged in the aged WT (Figure 5A, 5D) and decreased in the young IL-10^−/−^ mice (Figure 5E, 5H) with Los. ARB treatment reduced the levels of nitrotyrosine (Figure 6A–6B, 6F–6G, 6K–6L) and Nix (Figure 6A, 6C, 6F, 6H, 6K, 6M) in all groups. Relative mtDNA copy numbers decreased in aged WT and aged IL-10^−/−^ (Figure 6D) mice treated with Los (Figure 6N), but remained constant in young IL-10^−/−^ mice (Figure 6I). There was no change in the ATP levels in any of the treated groups (Figure 6E, 6J, 6O).

**Figure 7:**
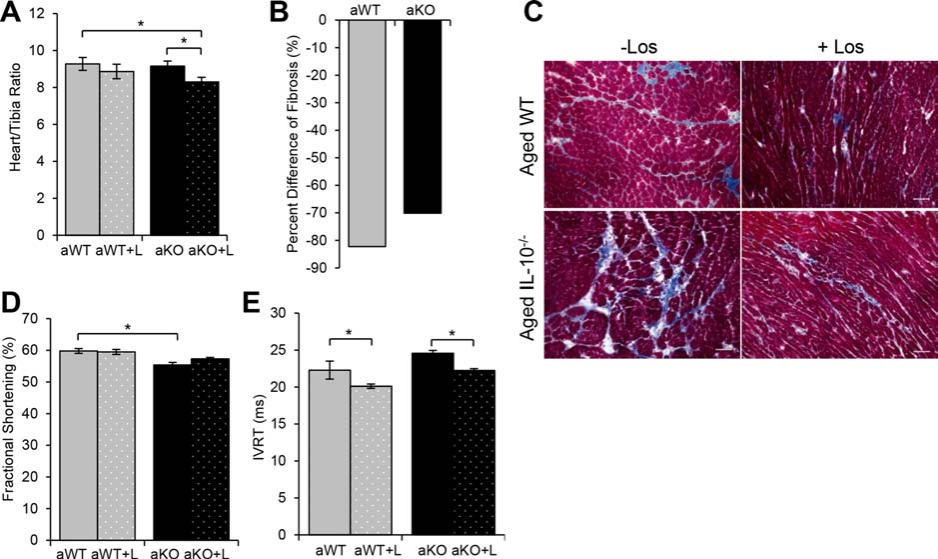
Effects of losartan treatment on cardiac remodeling and function **A.** Cardiac hypertrophy measured as a ratio of heart weight to tibia length of aged WT (aWT) mice, aWT with (+L) losartan treatment, aged IL-10^−/−^ (aKO) and aKO+L **B.** Quantification of fibrosis evident in Masson Trichrome stain **C.** Scale bar: 100 μm. **D.** Fractional shortening and **E.** isovolumetric relaxation time (IVRT) of aged WT and aged IL-10^−/−^ with (+L) and without losartan treatment. Data are means ± SEM *p < 0.05.

Phenotypically, AT_1_R blockade in the mouse model of the combined effect of aging and inflammation (aged IL-10^−/−^) was associated with less cardiac hypertrophy (Figure 7A) and with about 70% less fibrotic tissue (Figure 7B, 7C). In contrast, losartan had no effect on cardiac hypertrophy in the aged WT mice (Figure 7A); although, there was about 80% decrease of cardiac fibrosis (Figure 7B, 7C). Functionally, echocardiographic measurements revealed a reduction in fractional shortening in the aged IL-10^−/−^ as compared to the aged WT (Figure 7D and Supplemental Figure 1A). In contrast, isovolumetric relaxation time (IVRT) was shortened with Los in the aged WT and aged IL10^−/−^ mice (Figure 7E and Supplemental Figure 1B). Although aging and the combination of inflammation and aging both have up-regulated AT_1_R signaling, they each have unique downstream molecular pathways that converged in a similar pathohistological phenotype which was improved with ARB treatment.

## DISCUSSION

**Figure 8:**
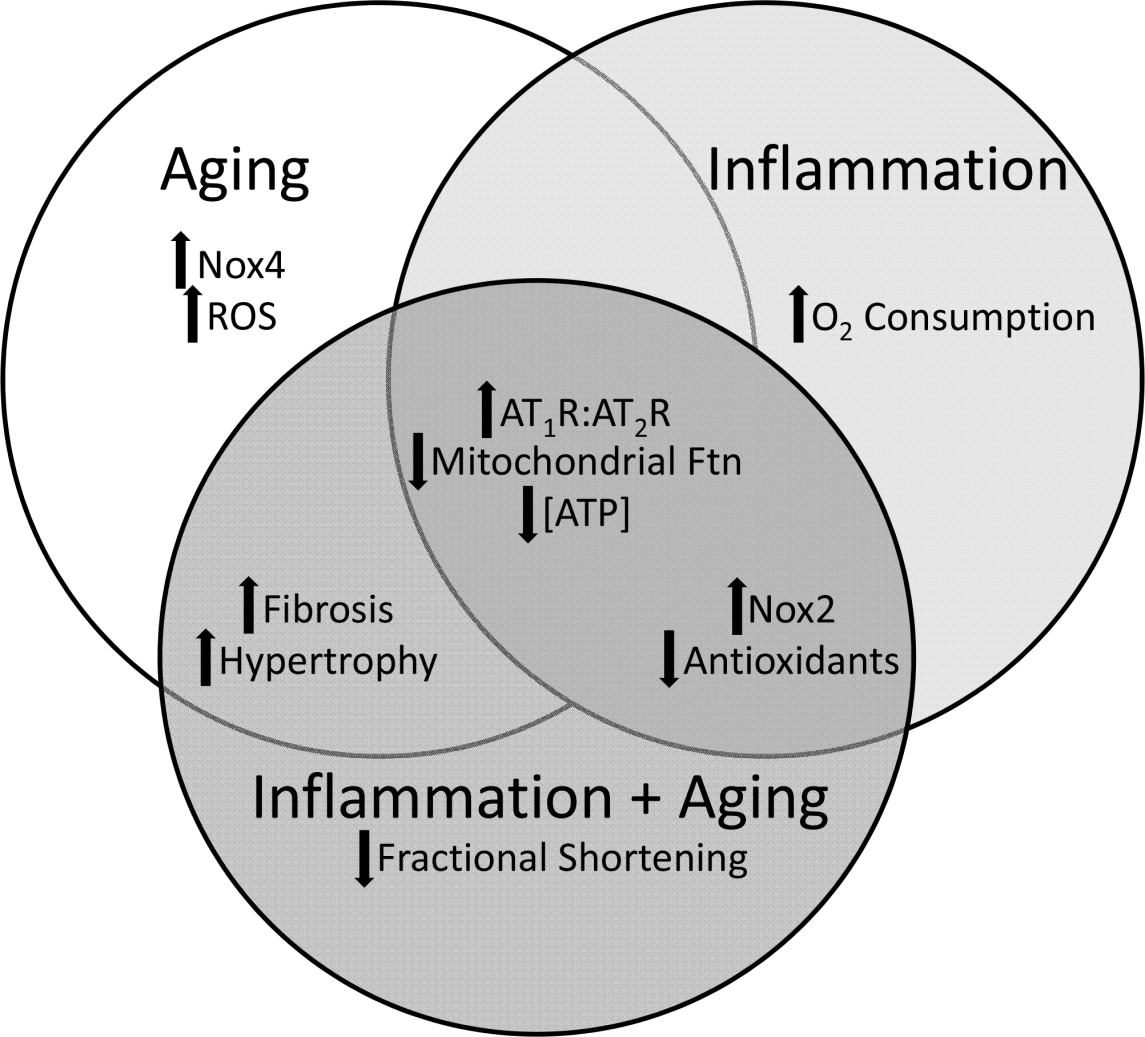
Similarities and differences in the molecular mechanisms and phenotypic characteristics of aging, inflammation, and the combination There are shared and distinct characteristics of aging, inflammation and the combined effect of aging and inflammation in the pathogenesis of cardiomyopathy.

Studies have shown an age-related cardiac hypertrophy and functional decline in older healthy individuals as well as in association with co-morbidities including frailty [9, 32]. Many studies have investigated the interface between RAS and mitochondrial health in the context of aging or inflammation but not the combination of both aging and inflammation. In this present study, we sought to determine the longitudinal effects of increased AT_1_R signaling in the pathological aging (combination of aging and inflammation) on cardiac muscle and mitochondria and how that compares to “normal” aging and inflammation independently (Figure 8). Moreover, conditions of chronic inflammation have been suggested to be premature or accelerated aging. However, our data suggests that aging and inflammation have distinct molecular mechanisms that in time will result in a similar phenotype such as cardiomyopathy.

AngII signaling through AT_1_R has been implicated in various pathological cardiac conditions. Aberrant AT_1_R signaling is known to mediate many deleterious effects of AngII in the cardiac muscle including myocyte hypertrophy and interstitial fibrosis [33, 34]. Transgenic mice over-expressing AT_1_R in cardiac myocytes developed cardiac hypertrophy and remodeling leading to a premature death caused by heart failure [35]. In our mouse models, we observed an increase in overall levels of AT_1_R in aged WT in comparison to the young WT. Interestingly, aged IL-10^−/−^ mice had lower AT_1_R levels than the aged WT, which may suggest a negative feedback inhibitory signal in the combinatory effect of aging and inflammation in an attempt to dampen down the pro-inflammatory AT_1_R. Previous data has shown that multiple molecules can modulate AT_1_R expression including IL-6, interferon-γ, tumor necrosis factor-α, insulin-like growth factor 1, nitric oxide and ROS [36]. All of these molecules are up-regulated in IL-10^−/−^ mice [37] and can provide potential explanations to the reduction in AT_1_R expression in the aged IL-10^−/−^ mice. Moreover, the regulation of AT_1_R can be modulated by AT_2_R and vice versa [38]. Given the reports documenting an inhibitory effect of AT_2_R on AT_1_R signaling, it is possible that the pathological portfolio observed can be driven by an overexpression of AT_1_R or under-expression of AT_2_R. Nonetheless, there was an overall increase in the AT_1_R:AT_2_R in aging, inflammation, and the combination that was restored with losartan treatment.

The differential activation of AT_1_R on Nox proteins is not fully elucidated. Interestingly, our data demonstrated a distinction on AT_1_R-driven expression of Nox enzymes in aging and inflammation. In the aging WT mice, we observed an increase in the expression of Nox4, which has been shown to be up-regulated during aging and in response to hypertrophic stimulation [39]. However, we did not observe an age-related increase in Nox4 expression in the aged IL-10^−/−^ mice. In contrast, both the young and aged IL-10^−/−^ mice expressed increased Nox2, which has been shown to be activated by agonists such as cytokines and growth factors unlike the constitutively active Nox4 [40]. Interestingly, the totality of this data suggest that in our mouse models, the influence of inflammation supersedes that of aging in the expression of Nox4 and further supports the notion that inflammatory conditions may be precipitated by a unique pathway activation and not necessarily a premature activation of aging pathways.

In further support of this notion, our data demonstrate a loss of redox balance that is driven in aging by increased generation of ROS while inflammation is characterized by diminished antioxidant levels. This distinction is consistent with our data on age-related increased expression of Nox4, a mitochondrial enzyme likely driving the increase in mitochondrial H_2_O_2_ evident in our aging WT mice. Moreover in our study, aging had no effect on the levels of antioxidants, in contrast to previous reports [41]. This discrepancy can be due to the differences in model organisms and/or experimental procedures. The combination of aging and inflammation resulted in the lowest levels of antioxidants, in agreement with the documented influence of circulating proinflammatory cytokines on antioxidant levels [42]. It is important to note that the use of ARBs can reduce ROS [44] and restore the balance between ROS and antioxidants independent of altering the antioxidant levels. Interestingly in this study, ARB treatment further decreased catalase expression in the inflammation model. We propose that losartan corrected the imbalance in ROS and antioxidants, negating the need and thus levels of catalase protein.

Mitochondria are particularly sensitive to oxidative damage, aging, and inflammation [45]. The high energy demand on the mitochondria in an inflamed environment, like that present in the young IL-10^−/−^ mice, likely drives the observed increase in oxygen consumption which did not result in increased ATP concentration and thereby suggests high ATP utilization [46]. The process of aging was associated with a greater reduction in oxygen consumption in the IL-10^−/−^ mice as compared to WT mice, likely due to the high burnout rate of mitochondria functioning at such an increased level for an extended period of time. Consequently, there is an increase in the number of damaged mitochondria in the aged WT and aged IL-10^−/−^ mice compared to the young counterparts. Treatment with losartan reduced the amount of Nix-expressing damaged mitochondria in all groups and consequently relative mtDNA numbers in aging and frailty possibly through the re-activation of autophagic processes.

Despite different precipitating factors, losartan was able to halt or even reverse adverse effects of increased AT_1_R and mitochondrial damage. Treatment of our mouse models with an ARB corrected the molecular changes evident with aging, inflammation, and the combined effect in the cardiac muscle. Furthermore, losartan treatment reduced fibrosis and shortened IVRT in aging and the combination of aging and inflammation. This data supports the notion that pathological changes may indeed be reversible. Additionally, this research highlights that the combined effect of aging and inflammation is molecularly distinct from that of aging and inflammation alone. Therefore, pinpointing the exact molecular changes associated with each may fine tune future interventions.

## MATERIALS AND METHODS

### Animals

All mouse protocols were approved by the Animal Care and Use Committee of Johns Hopkins University (JHU) School of Medicine. Male C57BL/6J (WT) and B6.129P2*-IL10^tm1Cgn/^*J** (IL-10^−/−^) fully backcrossed on C57BL/6 background [47] mice were born in the JHU colony to breeders purchased from Jackson Laboratory, and then housed in specific pathogen-free (SPF) barrier conditions and colonies were monitored for infection through one sentinel cage per rack. The mice are housed in SPF conditions to minimize the spontaneous development of generalized enterocolitis [48]. Moreover, IL-10^−/−^ mice on a C57Bl/6 background are less likely to spontaneously develop enterocolitis [49].

The mice are housed in said conditions until they reached the appropriate age for study [young (4–6 months) or aged (22–24 month)]. A subset of mice was subjected to losartan (0.6 g/Liter for young mice and 0.9 g/Liter for aged mice, Cozaar, Merck) *ad libitum* in their drinking water for 4 weeks. There were no significant changes in the body weight of the mice on losartan or placebo. After the time period of 4 weeks, the mice were sacrificed using an inhalation overdose of isoflurane (IsoFlo). The cardiac muscles were quickly excised and weighed. They were then processed accordingly for subsequent experiments.

### Histology/Immunofluorescence

A portion of the cardiac muscles were embedded in Tissue-Tek O.C.T. Compound (Sakura) and multiple thin sections (10 μm) were cut using a cryostat (Microm). Subsequently, the sections were stained with hematoxylin and eosin (H&E), Masson's Trichrome (Polysciences, Inc.) or using immunofluorescence techniques. Masson's Trichrome staining was carried out according to the manufacturer protocol with the addition of an one hour 10% formalin fix at room temperature (RT) prior to the fixation in Bouin's solution [50]. For immunostaining, the sections were fixed with 4% paraformaldehyde for 15 minutes at RT then blocked with 5% BSA/0.3% TritonX-100/PBS for one hour at RT, incubated with the primary antibody, 8-oxoG DNA Lesion (Santa Cruz), overnight at 4°C and incubated with secondary AlexFluor antibody (Invitrogen) at RT for 1 hour. Slides were mounted with Vectashield Hard Set with Dapi (Vector Laboratories). All images were taken with an Eclipse N*i* microscope (Nikon).

### Morphometry

The amount of fibrosis and the cardiac muscle cross-sectional area (CSA) was determined using images obtained from the Masson's Trichrome stain using Nikon Nis-Elements 4.20 software. The percentage of fibrosis was then calculated by dividing the total areas of fibrosis by the CSA. The intensity of the 8-oxoG DNA Lesion immunostain was measured using Nikon Nis-Elements 4.20 software.

### Mitochondrial isolation

Mitochondria were isolated from approximately 60 mg of fresh cardiac muscle using a standard protocol [51] adjusted for the smaller amount of tissue. BCA assay (Pierce) was used to determine the protein concentration of the purified mitochondria.

### Measurement of reactive oxygen species (H_2_O_2_)

The amount of H_2_O_2_ emitted from isolated mitochondria was determined using the Amplex Red Hydrogen Peroxide/Peroxidase Assay kit (Invitrogen) using the protocol supplied by the manufacturer for measuring H_2_O_2_ released by cells, with minor modifications: 30 μg of fresh mitochondria were used in a total volume of 20 μL. The reaction buffer contained 225 mM mannitol, 75 mM sucrose, 10 mM Tris, 10 mM K_2_HP_4_, 0.1 mM EDTA, 0.08 mM MgCl_2_, and 0.2% BSA, pH 7.1 and 5 mM succinate was used as the activator [52].

### Measurement of peroxidase activity

The peroxidase activity of isolated mitochondria was determined using the Amplex Red Hydrogen Peroxide/Peroxidase Assay kit (Invitrogen) using the protocol supplied by the manufacturer. Thirty μg of fresh mitochondria were used in a total volume of 50 μL.

### Protein extraction/western blot analysis

Protein was extracted from flash frozen cardiac muscles using T-PER (Thermo Scientific) with the addition of Protease (Complete Mini, Roche) and Phosphatase (PhosStop, Roche) inhibitors. Equal concentrations of protein were electrophoresed using Bis-Tris gels (Invitrogen) and transferred onto a nitrocellulose membrane. Membranes were incubated with primary antibodies overnight at 4°C. The following primary antibodies were used: Nox2, Catalase (Abcam), Nox4 (Novus), Nitrotyrosine (Millipore), Nix (Invitrogen), and Actin (Sigma). AT_1_R and AT_2_R antibodies used were purchased from Santa Cruz and previously validated for specificity in our laboratory and others [53–55]. HRP-conjugated secondary antibodies were used to detect bands (Amersham). Quantitative Western blot analyses were performed using ImageJ (National Institutes of Health).

### Oxygen consumption

For monitoring respiration, mitochondria were isolated from fresh cardiac tissue as previously described [56]. Ten micrograms of mitochondria were aliquoted into the wells of a polyethyleneimine-coated XF96 cell culture 96-well microplate (Seahorse Bioscience). Mitochondria were placed in 300 μL of mitochondrial buffer [20 mM Hepes, 137 mM KCl, 2.5 mM MgCl2, 2 mM K2HPO4, 0.5 mM EGTA, and 0.2% (wt/vol) BSA (pH 7.3)]. All experiments were performed at 37 °C. The oxygen consumption rates were determined by using a compartment model-based deconvolution algorithm [53].

### Transmission electron microscopy

Thin slices of fresh cardiac muscle were fixed overnight in 2% glutaraldehyde, 0.1 M cacodylate buffer, 3% sucrose and 3 mM CaCl_2_ at 4^o^C with agitation. Fixed tissues were subjected to 1% osmium tetroxide reduced in potassium ferrocyanide for 1 hour and stained *en bloc* with 2% aqueous uranyl acetate and serially dehydrated in graded ethanol and propylene oxide. Prepared samples were embedded in Eponate 12 Resin (Ted Pella). Multiple thin sections (70–90 nm) were cut for each sample on a Reichert-Jung Ultracut E microtome, placed on 200 mesh copper grids, stained with uranyl acetate and lead citrate, and viewed on a Hitachi 7600 TEM (Hitachi High Technologies America, Inc) with an AMT Advanced Microscopy Techniques digital camera (Danvers, MA).

### Mitochondria biogenesis

DNA was extracted from flash frozen cardiac muscle using DNeasy (Qiagen). PCR amplification was performed with a Stratagene mx3000p machine. Primers included cytochrome B forward: 5′-TATTCCTTCATGTCGGACGA-3′, cytochrome B reverse: 5′-AAATGCTGTGGCTATGACTG-3′, genomic GAPDH forward: 5′-ATGTTTGTGATGGGTGTGAA-3′ and genomic GAPDH reverse: 5′-ATGCCAAAGTTGTCATGGAT-3′ [57].

### ATP concentration

ATP was extracted from 10 μg of flash frozen cardiac muscle. Ice cold 0.4 M perchloric acid was added to the cardiac tissue and it was immediately homogenized using the Bullet Blender 24 (Next Advance) at 4°C. The reaction was neutralized with ice cold 2 M KHCO_3_ [58]. The extracted ATP was used with an ATP Bioluminescent Assay Kit (Sigma) according to the manufacturer's protocol and measured using Wallac Victor2, 1420 Multilabel Counter (PerkinElmer) [37].

### Cardiac ultrasound

Trans-thoracic echocardiography and Tissue Doppler Imaging (TDI) were performed in non-sedated mice treated with losartan for 8 weeks using the Vevo 2100 high-resolution micro-imaging ultrasound system, equipped with a 40-MHz linear micro scan transducer (Vesualsonics, Toronto, ON, Canada) by a single sonographer blinded to the study. The two-dimensional and M-mode echocardiogram were obtained in the parasternal short and long axis view of the left ventricle (LV) at the level of the papillary muscles and at sweep speed of 200 mm/sec. The M-mode echocardiogram images were used to measure the LV chamber diameter at end of diastole (LVEDD) and LV chamber diameter at end of systole (LVESD); these measurements were used to calculate the fractional shortening. From the apical four-chamber view, TDI was used to measure the isovolumetric relaxation time (IVRT) at the septal mitral annulus level. Three to five measurements were performed according to the recommendations set by the American Society of Echocardiography [59]

### Statistical analysis

All values are expressed as a mean ± s.e.m. Significance was set at *p* ≤ 0.05 and determined by either unpaired Student's *t*-test or two-way Analysis of Variance (ANOVA) followed by the Tukey test.

## SUPPLEMENTARY FIGURE


