# Supplementary material for: Combined effects of aging and inflammation on renin-angiotensin system mediate mitochondrial dysfunction and phenotypic changes in cardiomyopathies
Source: Oncotarget. 2015 May 18;6(14):11979–93. doi: 10.18632/oncotarget.3979 (PMC4494917; doi:10.18632/oncotarget.3979)
Supplement: Supplementary file 1 [file oncotarget-06-11979-s001.pdf]

SUPPLEMENTARY FIGURE

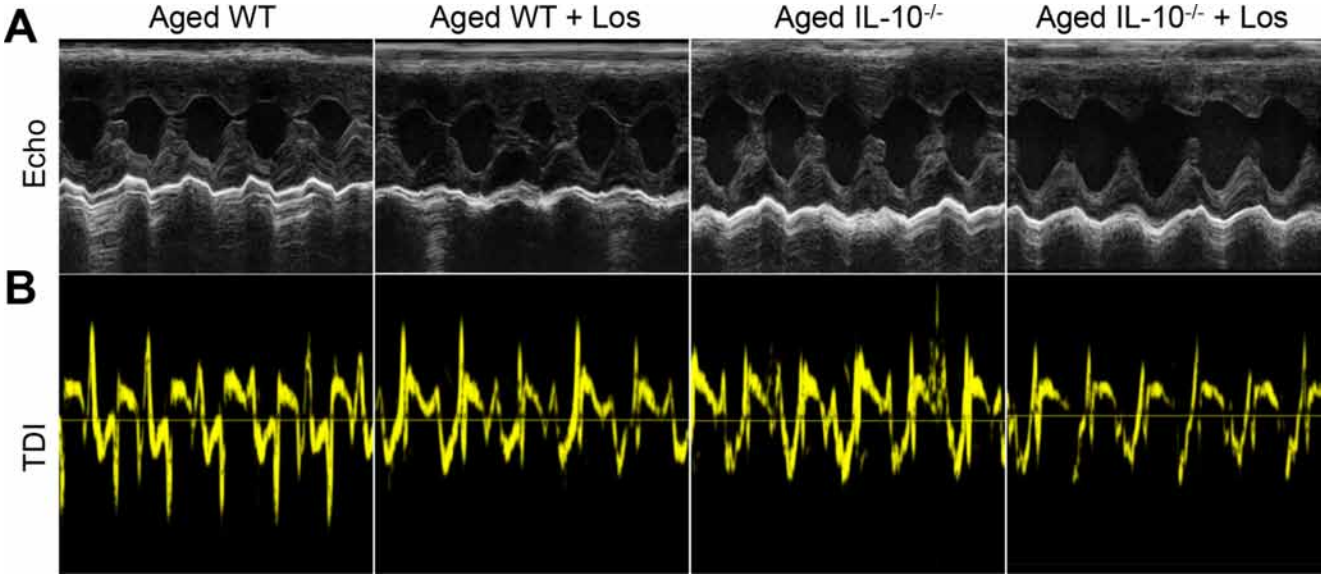

Supplementary Figure S1: Representative images of cardiac function with and without Losartan treatment. A. Echocardiography (Echo) and **B.** Tissue Doppler Images (TDI) of aged Wild-type (WT) and aged IL-10$^{-/-}$ mice with (+Los) or without losartan treatment.
